# A Population-Based Study on the Association between Benign Prostatic Enlargement and Rheumatoid Arthritis

**DOI:** 10.1371/journal.pone.0133013

**Published:** 2015-07-14

**Authors:** Ya-Mei Tzeng, Li-Ting Kao, Herng-Ching Lin, Chao-Yuan Huang

**Affiliations:** 1 Graduate Institute of Life Science, National Defense Medical Center, Taipei, Taiwan; 2 Sleep Research Center, Taipei Medical University Hospital, Taipei, Taiwan; 3 School of Public Health, National Defense Medical Center, Taipei, Taiwan; 4 Department of Urology, National Taiwan University Hospital, College of Medicine National Taiwan University, Taipei, Taiwan; 5 School of Public Health, Taipei Medical University, Taipei, Taiwan; Northwestern University, UNITED STATES

## Abstract

Benign prostatic hyperplasia is one of the chronic inflammatory conditions in ageing male populations. Rheumatoid arthritis (RA) is a major autoimmune disease and is also regarded as a chronic inflammatory disorder. Although RA and benign prostatic enlargement (BPE) may share the same underlying etiologies, almost no study has ever attempted to explore the relationship between RA and BPE. The aim of this study was to explore the relationship between RA and BPE using a population-based dataset. This case-control study used data retrieved from the Taiwan Longitudinal Health Insurance Database 2005. This study comprised 18,716 patients with BPE and 18,716 age-matched patients without BPE. Conditional logistic regression analyses were performed to calculate the odds ratio (OR) for having been previously diagnosed with RA between patients with BPE and comparison patients. In total, 485 of the 37,432 sampled patients (1.3%) had received a prior RA diagnosis. There was a significant difference in the prevalence of prior RA between cases and controls (1.6% vs. 1.0%, *p*<0.001). After adjusting for patient’s urbanization level, monthly income, geographic region, and obesity, the adjusted OR was 1.54 (95% CI = 1.28~1.85) for patients with BPE compared to comparison patients. In addition, the sensitivity analysis showed that BPE was consistently and significantly associated with a prior RA diagnosis even after excluding subjects diagnosed with RA within 1, 2, or 3 years prior to the index date (the adjusted ORs were 1.46, 1.50, and 1.42, respectively). We concluded that there was a significant association between prior RA and BPE. Further large-scale longitudinal studies are suggested to clarify the causal relationship between RA and BPE.

## Introduction

Benign prostatic hyperplasia (BPH) is a common chronic condition in ageing male populations. Although the mechanism contributing to BPH remains unclear, chronic inflammation was suggested to play an important role in its pathogenesis and development [1–3]. Nickel even demonstrated that inflammation in the prostate gland appears to more closely related to BPH than to the clinical syndrome of chronic prostatitis [4]. An inflammatory condition may result in a large prostate and a high serum prostate-specific antigen level [3].

Rheumatoid arthritis (RA) is a major autoimmune disease which is considered a chronic inflammatory disorder. Studies also suggested that some cytokines and their products may play significant roles in the pathogenesis of RA [5, 6]. However, although RA and BPE may share the same underlying etiologies, almost no study has ever attempted to explore the relationship between RA and BPE. According to our knowledge, only one study by Koskimaki et al. ever investigated the association of RA with lower urinary tract symptoms (LUTSs), but they failed to establish any association [7]. Therefore, the aim of this study was to explore the relationship between RA and benign prostatic enlargement (BPE) using a population-based dataset study.

## Methods

### Database

This case-control study used data retrieved from the Longitudinal Health Insurance Database 2005 (LHID2005). The LHID2005, which is derived from medical claims records of the Taiwan National Health Insurance (NHI) program, includes original medical claims and registration files for 1,000,000 enrollees under the Taiwan NHI program. The Taiwan National Health Institute randomly selected these 1,000,000 enrollees from all enrollees listed in the 2000 Registry of Beneficiaries (*n* = 23.72 million). Prior studies and the Taiwan National Health Institute have demonstrated the high validity of data derived from the NHI program [8]. The LHID2005, which was open to the researchers in Taiwan, was available from the National Health Research Institutes (http://nhird.nhri.org.tw/date_01.html).

This study was exempt from full review by the Institutional Review Board of the National Defense Medical Center because the LHID2005 consists of de-identified secondary data released to the public for research purposes. This study did not have written informed consent. All patient records/information was anonymized and de-identified prior to analysis.

### Selection of cases and controls

For cases, we first identified 18,937 patients who had received a first-time BPE diagnosis (ICD-9-CM code 600.0) between January 1, 2009 and December 31, 2012 (*n* = 31,204). In Taiwan, a physician makes a diagnosis of BPE on an outpatient basis must include digital rectal examination and clinical symptoms reported by patients. We then excluded 221 patients aged younger than 40 years because the prevalence of BPE is very low in this age group. Ultimately, 18,716 patients with BPE were included as cases in this study. Furthermore, we defined the date of receiving their first-time BPE diagnosis as the index date.

We likewise selected controls from the remaining enrollees of the LHID2000. We excluded those subjects who had ever received a BPE diagnosis since initiation of the NHI program in 1995. We then randomly selected 18,716 controls (one control per case) to match the cases by age and index year. In this study, the year of the index date for cases was defined as the year in which the cases received their first BPE diagnosis. However, for controls, the index year was simply a matched year in which the controls had a healthcare utilization. Furthermore, we defined the first healthcare utilization occurring in the index year as the index date for controls.

### Exposure assessment

This study identified RA cases based on ICD-9-CM code 714.0. In Taiwan, RA is usually diagnosed by rheumatologists. In order to increase the validity of the RA diagnoses, only those patients who had received two or more RA diagnoses prior to the index date, with at least one being made by a rheumatologist, were included in this study. In addition, we limited RA cases to those who had been prescribed at least one type of disease-modifying antirheumatic drug prior to the index date in order to increase the diagnostic validity.

### Statistical analysis

The SAS system for Windows (vers. 8.2, SAS Institute, Cary, NC) was used to perform all statistical analyses in this study. We used Chi-squared tests to explore differences in sociodemographic characteristics between cases and controls. We further performed conditional logistic regression analyses (conditioned on age group and index year) to calculate the odds ratio (OR) and corresponding 95% confidence interval (CI) for having been previously diagnosed with RA between cases and controls. The conventional *p*≤0.05 was used to assess statistical significance.

## Results

Mean age of cases and controls was 51.3 ± 15.3 (± standard deviation). After matching for age and index date, Table 1 shows that there were significant differences in geographic region, monthly income, urbanization level, and obesity between cases and controls (all *p*<0.001).

**Table 1:** Demographic characteristics of patients with benign prostatic enlargement (BPE) and controls in Taiwan (n = 37,432).

Variable	Patients with BPE (*n* = 18,716)		Controls (*n* = 18,716)		*p* value
	Total no.	Percent (%)	Total no.	Percent (%)	
Urbanization level					<0.001
1 (most urbanized)	5617	30.0	5067	27.1	
2	5176	27.7	5054	27.0	
3	2748	14.7	2954	15.8	
4	2793	14.9	2970	15.9	
5 (least urbanized)	2382	12.7	2671	14.2	
Monthly income					<0.001
≤ NT15,840	7856	42.0	7640	40.8	
NT$15,841~25,000	6227	33.3	6868	36.7	
≥ NT$25,001	4633	24.8	4208	22.5	
Geographic region					<0.001
Northern	8822	47.1	8244	44.1	
Central	4580	24.5	4545	24.3	
Southern	4882	26.1	5403	28.9	
Eastern	432	2.3	524	2.8	
Obesity	182	1.0	106	0.6	<0.001

The average exchange rate in 2012 was US$1.00≈New Taiwan (NT)$29.3.

Table 2 presents the prevalence of prior RA between cases and controls. In total, 485 of the 37,432 sampled patients (1.3%) had received an RA diagnosis before the index date. Chi-squared test shows that there was a significant difference in the prevalence of prior RA between cases and controls (1.6% vs. 1.0%, *p*<0.001). We further found that patients with RA had averagely earlier onset age of BPH than patients with RA (66.8 ± 11.8 vs. 68.6 ± 10.9, *p* = 0.008).

**Table 2:** Prevalence and odds ratios (ORs) for prior rheumatoid arthritis (RA) among sampled subjects.

Presence of prior RA	Total (*n =* 37,432)		Patients with BPE (*n =* 18,716)		Controls (*n =* 18,716)	
	*n*, Percent (%)		*n*, Percent (%)		*n*, Percent (%)	
Yes	485	1.3	292	1.6	193	1.0
Crude OR (95% CI)	—		1.52*** (1.27–1.83)		1.00	
Adjusted OR (95% CI) ^a^	—		1.54*** (1.28–1.85)		1.00	

Notes: The OR was calculated by a conditional logistic regression which was conditioned on age group and index year; BPE, benign prostatic enlargement

*** p<0.001.

^a^ Adjustments were made for patient’s urbanization level, monthly income, geographic region, and obesity.

The ORs and its corresponding 95% CI for having been previously diagnosed with RA between cases and controls were also presented in Table 2. The conditional logistic regression (conditioned on age and index year) suggested that the OR of prior RA for cases was 1.52 (95% CI: 1.27~1.83, *p*<0.001) compared to controls. We further found that the OR of having previously received an RA diagnosis among cases was 1.54 (95% CI: 1.28~1.85; *p*<0.001) that of controls after adjusting for geographic region, monthly income, urbanization level and obesity.

In addition, the sensitivity analysis of the relationship between BPE and prior RA is presented in Table 3. It showed that BPE was consistently and significantly associated with a prior RA diagnosis even after excluding subjects diagnosed with RA within 1, 2, or 3 years prior to the index date (the adjusted ORs were 1.46, 1.50, and 1.42, respectively).

**Table 3:** Sensitivity analysis.

Outcome variable	Excluding patients who received a RA diagnosis within 1 year prior to the index date		Excluding patients who received a RA diagnosis within 2 years prior to the index date		Excluding patients who received a RA diagnosis within 3 years prior to the index date	
	Patients with BPE	Controls	Patients with BPE	Control	Patients with BPE	Control
	N (%)					
Presence of prior RA						
Yes	259 (1.39)	181 (0.97)	232 (1.24)	158 (0.85)	215 (1.15)	154 (0.82)
Crude OR (95% CI)	1.44*** (1.19~1.74)		1.48*** (1.21~1.81)		1.40*** (1.14~1.73)	
Adjusted OR ^a^ (95% CI)	1.46*** (1.20~1.76)		1.50*** (1.22~1.83)		1.42*** (1.15~1.75)	

CI, confidence interval; OR, odds ratio; the OR was calculated by a conditional logistic regression which was conditioned on age group and index year.

*** p<0.001

^a^ Adjusted for patient’s urbanization level, monthly income, geographic region, and obesity.

## Discussion

To our knowledge, this is the first population-based study to investigate the association between RA and BPE. We found that there was a significant relationship between RA and BPE (adjusted OR = 1.54) even after adjusting for potential confounding factors. However, one study investigated 1963 men aged ≥50 years with LUTSs, and after adjusting for age and other non-urologic diseases, it failed to establish a significant association between RA and LUTSs [7].

Although the mechanism of how RA leads to BPE still remains unclear, it might be explained by the inflammatory condition of RA. The prior literature documented an association between RA and inflammation and reported that RA is associated with an elevated production of inflammatory factors, such as tumor necrosis factor (TNF)-α, interleukin (IL)-1, IL-6, IL-8, and transforming growth factor (TGF)-β. Moreover, those studies showed that TNF-α might be the major cytokine included in the inflammatory mechanism, and IL-1 is a key mediator in regard to the RA symptoms, including cartilage and bone destruction [9–11].

Additionally, previous studies mentioned that an inflammatory response in BPE might develop from abnormal immune function. For example, one study found that inflammatory factors might stimulate the proliferation of BPH-derived prostate stromal cells [2]. Furthermore, various studies also demonstrated that inflammatory factors might cause prostatic tissue injury and induce the production of prostatic proliferating cells in BPH [12, 13]. Therefore, RA and BPE might share similar underlying inflammatory mechanisms. Accordingly, it is plausible that the chronic inflammatory condition associated with RA might contribute to the development and exacerbation of BPE.

Our study was based on a large population-based dataset, and its strength was that the NHI program has only a single-payer system and covers wide health benefits in Taiwan. The specific design ensured that analysis had sufficient statistical power and provided a sufficient sample size. There are still several limitations to our study. First, the LHID2005 supplies no laboratory data, and this did not allow us to explore the association between RA and BPE. Second, the dataset used in this study contained no information on health behaviors such as diet, nicotine and alcohol consumption, and the level of education, which might be the potential risk factors for RA or BPE. Third, our study only included the largely ethnic Chinese population in Taiwan, so that the ability to generalize the results to other countries might be low. Finally, there may be surveillance bias as patients with RA have a great tendency to have more frequent outpatient clinic visits, which may lead to early detection of BPE. However, we further examined the number of outpatient visits for urological services within one year prior to index date, and we found that there was no significant difference in the number of outpatient visits for urological services between cases and control (0.75 vs. 0.72, *p* = 0.326). Therefore, the possible surveillance bias may not compromise the findings of this study.

In conclusion, our study found a significant association between prior RA and BPE. We suggest that physicians should be alert to this relationship and provide routine urologic examinations of patients with RA. Nevertheless, further large-scale longitudinal studies are suggested to clarify the causal relationship between RA and BPE.
